# Country Holidays for Town Children

**Published:** 1889-06-29

**Authors:** 


### COUNTRY HOLIDAYS FOR TOWN CHILDREN.

The Ragged School Union has started a Holiday Homes Fund, and issues as an appeal a clever and characteristic pamphlet. There are pictures of children in a London garret, and again in a country meadow, and such poetical headings as "Drooping Cage Birds" attract the attention of the sentimentally inclined. Yet, sentiment apart, there can be no doubt that of all modern movements the country holiday is not the least. Without health, without happiness, no life can be of great worth, and health and happiness are best given a street arab by a fortnight amongst the wonders of field and lane. The pure air, the simple country ways, the wholesome diet, all serve to give a healthy tone to both mind and body. All success to the various associations which thus benefit little children!

According to the present arrangements of the Ragged School Union, five homes will be opened shortly, two of them, however, having been open all winter for the reception of special cases requiring immediate change. According to plans now in progress, the homes this year will be: Broadstairs and Folkestone, for those ordered sea breezes; Thursley, on the breezy common beyond Godalming; East Grinstead, a famous health resort; Brenchley, among the Kentish hop-gardens, and specially for elder scholars; and Caterham, in the well-known Caterham Valley. In addition, many friends are arranging to co-operate, as in past years, by undertaking the oversight of the children sent to the care of cottagers in many parts, such as Bletchley, Bushey Heath, Brighton, Chislehurst, Salisbury, Elstree, Halstead, etc.

The Poor Children's Aid Society, which the *Globe* takes under its special protection, is issuing an appeal for funds needed to reopen the country home it started last year. Five shillings will maintain a child at this home for a week, and surely those who tasted the joys of a country holiday last year will have the pleasure renewed this summer.

In the *Sunday at Home* there is a plea for convalescent children discharged from the hospitals, but not admissable to ordinary homes. The writer says: "Many small invalids have to be sent away from the hospitals without any chance of an intermediate resting-place. From the comfort of the wards the most of them must go straight to the wretchedness which is awaiting at home; but a few weeks spent in fresh air, with careful tendance and cleanliness and wholesome food would brace them up into a more fitting condition of mind and body for traversing the hard path which their little feet must follow. The kindly help given last year encourages a hope for increased assistance this season. Alas! for the little creatures who have never seen the country, and who have seldom known either kindness or comfort. How sad, even in health, but in sickness and weakness well-nigh unbearable."

Then there is the central institution in Buckingham-street, about which we hope to say more on another occasion, as they have not yet sent us their report. So great is the benefit that this country holidays movement can work, that we mean to keep it prominently before our readers all the summer, and not let any of them take a holiday with an easy conscience unless they have first provided a holiday for some poor street arab. Five shillings is such a very small sum!

